# Comparative Transcriptomic Analysis of Genes in the 20-Hydroxyecdysone Biosynthesis in the Fern *Microsorum scolopendria* towards Challenges with Foliar Application of Chitosan

**DOI:** 10.3390/ijms24032397

**Published:** 2023-01-25

**Authors:** Siriporn Sripinyowanich, Sahanat Petchsri, Pumipat Tongyoo, Taek-Kyun Lee, Sukchan Lee, Won Kyong Cho

**Affiliations:** 1 Department of Botany, Faculty of Liberal Arts and Science, Kasetsart University, Kamphaeng Saen Campus, Nakhon Pathom 73140, Thailand; 2 Center for Agricultural Biotechnology, Kasetsart University, Kamphaeng Saen Campus, Nakhon Pathom 73140, Thailand; 3 Center of Excellence on Agricultural Biotechnology: (AG-BIO/MHESI), Bangkok 10900, Thailand; 4 Risk Assessment Research Center, Korea Institute of Ocean Science & Technology, Geoje 53201, Republic of Korea; 5 Department of Integrative Biotechnology, College of Biotechnology and Bioengineering, Sungkyunkwan University, Suwon 16419, Republic of Korea; 6 College of Biotechnology and Bioengineering, Sungkyunkwan University, Suwon 16419, Republic of Korea

**Keywords:** chitosan, differential expressed gene, *Microsorum scolopendria*, transcriptome, 20-hydroxyecdysone

## Abstract

*Microsorum scolopendria* is an important medicinal plant that belongs to the Polypodiaceae family. In this study, we analyzed the effects of foliar spraying of chitosan on growth promotion and 20-hydroxyecdysone (20E) production in *M. scolopendria*. Treatment with chitosan at a concentration of 50 mg/L in both young and mature sterile fronds induced the highest increase in the amount of accumulated 20E. Using RNA sequencing, we identified 3552 differentially expressed genes (DEGs) in response to chitosan treatment. The identified DEGs were associated with 236 metabolic pathways. We identified several DEGs involved in the terpenoid and steroid biosynthetic pathways that might be associated with secondary metabolite 20E biosynthesis. Eight upregulated genes involved in cholesterol and phytosterol biosynthetic pathway, five upregulated genes related to the methylerythritol 4-phosphate (MEP) and mevalonate (MVA) pathways, and several DEGs that are members of cytochrome P450s and ABC transporters were identified. Quantitative real-time RT-PCR confirmed the results of RNA-sequencing. Taken together, we showed that chitosan treatment increased plant dry weight and 20E accumulation in *M. scolopendria*. RNA-sequencing and DEG analyses revealed key enzymes that might be related to the production of the secondary metabolite 20E in *M. scolopendria*.

## 1. Introduction

*Microsorum scolopendria* commonly known as “wart fern” is a valuable air-purifying ornamental plant and an important medicinal plant belonging to the Polypodiaceae family [1]. In traditional Polynesian medicine, *M. scolopendria* fronds are used as antioxidants and as medication for numerous physical and mental disorders [2,3]. The adaptogenic and anabolic effects of *M. scolopendria* demonstrated by modern pharmacological research are attributed to 20-hydroxyecdysone (20E), a non-androgenic steroid used as an ingredient in dietary supplements [4,5] to increase energy and muscle mass [6]. *M. scolopendria* fronds and 20E exert a neuroprotective effect in aging rats [2,6], improve cognitive impairment [7], protect against brain injury, and inhibit the production of reactive oxygen species (ROS) [8]. Additionally, it is currently being explored as a possible anti-carcinoma and anti-tumor agent in the human oral epithelium and breast [9,10,11]. Given its cellular target, 20E molecules have recently been hypothesized to adequately improve respiratory function and prevent the appearance of severe forms of COVID-19; a clinical trial is underway [6,12].

Several previous studies have shown that 20E is biosynthesized from cholesterol via 7-dehydrocholesterol and 3β, 14α-dihydroxy-5β-cholest-7-en-6-one (5β-ketodiol) [13,14]. Cholesterol is converted to 5β-ketone with the migration of hydrogen from the C-6 to the C-5 position. These findings, along with the previous observation that the ketone is efficiently converted to 20E, strongly suggest that the 5β-ketone is an intermediate formed immediately after cholesterol during the 20E biosynthesis [14]. 

Chitosan treatment markedly modulated the ratio of individual sterols, including an increase in cholesterol content (65%), a 2-fold increase in sitosterol, induction of stigmasterol levels by up to 26%, and a decrease in sitosterol content (35%) [15]. Elicitation with chitosan, a polysaccharide constituting an element of the fungal cell wall, influences sterol content [15] and triggers a set of genes involved in the production of secondary metabolites [16,17,18]. Over the past several decades, chitosan has been proven to improve plant production as a plant growth regulator and is assumed to control membrane fluidity [19,20] and permeability or serve as a precursor of plant steroids [15,21] involved in plant defense against fungal attacks in various plant cells [22,23,24]. 

To the best of our knowledge, only a few studies have investigated the effects of foliar-applied chitosan on plant sterol production, and none have focused on 20E biosynthesis, especially in *M. scolopendria*. In this study, we investigated the effects of chitosan treatment on plant growth and 20E accumulation. Furthermore, we identified several DEGs involved in metabolic pathway signaling linked to the synthesis of the secondary metabolite, 20E. The identified key genes associated with chitosan signaling and 20E production in *M. scolopendria* could be useful for further genetic analysis of 20E biosynthesis and pathway engineering.

## 2. Results

### 2.1. Effect of Chitosan on Growth of M. scolopendria 

**Figure 1:**
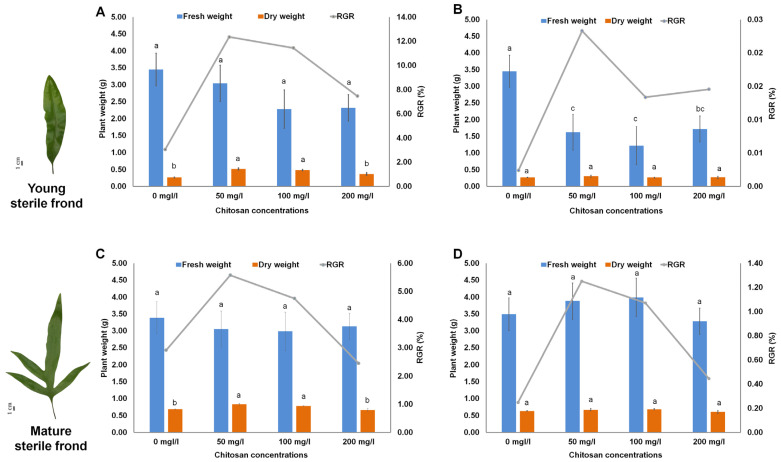
Influences of foliar chitosan application on growth of *Microsorum scolopendria* by measuring fresh weight and dry weight of young sterile fronds at 7 days after treatment (DAT) (**A**) and 14 DAT (**B**) and mature sterile fronds at 7 DAT (**C**) and 14 DAT (**D**). Data are presented as means ± standard error of the mean (SE) (n = 3). Different letters on top of bars indicate a significant difference (*p* ≤ 0.05) according to Duncan’s multiple range test.

In the present study, we used chitosan as an elicitor to reveal the molecular effects of chitosan in stimulating the synthesis of secondary metabolites in *M. scolopendria* fronds [25]. The fronds of *M. scolopendria* were sprayed with chitosan at three different concentrations, to test the effect of chitosan on plant growth promotion. After chitosan treatment, foliar application of chitosan positively affected the growth of *M. scolopendria* by increasing the dried mass of the fronds. However, the fern fronds treated with chitosan had slightly lower fresh weight than that of the control, whereas the chitosan-treated plants showed increased dry weight 7–14 days after treatment (DAT) compared with that of the control (Figure 1). The plants sprayed with chitosan at 50 mg/mL exhibited the highest dry weight in both young (1.93 times) and mature (1.21 times) sterile fronds after 7 DAT compared with that of the control (Figure 1). Specifically, fern plants sprayed with chitosan at 50 and 100 mg/mL significantly enhanced the growth rate of young sterile fronds at 7 DAT (by 12.3% and 11.4%, respectively) when compared with that at 0 DAT, whereas the growth rate of mature sterile fronds slightly increased by 5.6% and 4.8%, respectively. However, the effect of 200 mg/mL chitosan application did not significantly increase the relative growth rate (RGR), which was only slightly enhanced by 0.84 times in the mature sterile fronds when compared with that of the control (Figure 1). These data indicated that plants treated with chitosan at concentrations of 50 and 100 mg/mL showed increased growth compared with that in the control based on dried mass accumulation. Of the three different chitosan concentrations, chitosan treatment at 50 mg/mL showed the highest value for dried mass. We used 50 mg/mL as the optimal concentration for further experiments.

### 2.2. 20E Content Increased in Sterile Fronds of Chitosan-Treated M. scolopendria

**Figure 2:**
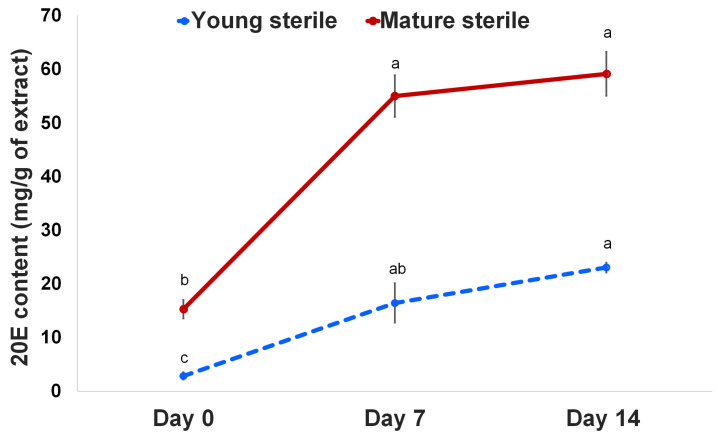
20-hydroxyecdysone (20E) content in young and mature sterile fronds of *M. scolopendria* after treatment with 50 mg/mL chitosan at 7 DAT and 14 DAT, respectively. Values within a row followed by the same superscript letter(s) are not significantly different at *p* ≤ 0.05 based on Duncan’s multiple range test.

Regarding plant growth and its attributes, chitosan elicits the activation of enzymes in the metabolic pathway linked with numerous secondary metabolites. Our previous study showed 20E accumulation in different frond tissues of the fern *Microsorum*; however, sterile fronds showed the highest 20E accumulation [26]. Thus, we focused on the application of chitosan to elucidate 20E and the associated mechanisms affecting plant growth and 20E biosynthesis. Our results showed that the plants treated with 50 mg/mL chitosan had significantly higher 20E production in young and mature sterile fronds compared with that in pre-treated fronds. As shown in Figure 2, the highest 20E production (59.14 mg/g) was observed in the mature sterile fronds at 14 DAT. However, the 20E content in the young and mature sterile fronds increased 5.79 and 3.60 times at 7 DAT and 8.11 and 3.87 times at 14 DAT, respectively, compared with that on 0 DAT (Figure 2). Therefore, foliar spraying of 50 mg/mL chitosan enhanced the 20E content in both young and mature sterile fronds. 

### 2.3. Identification of Differential Expressed Genes (DEGs) of M. scolopendria in Response to Chitosan Treatment

**Figure 3:**
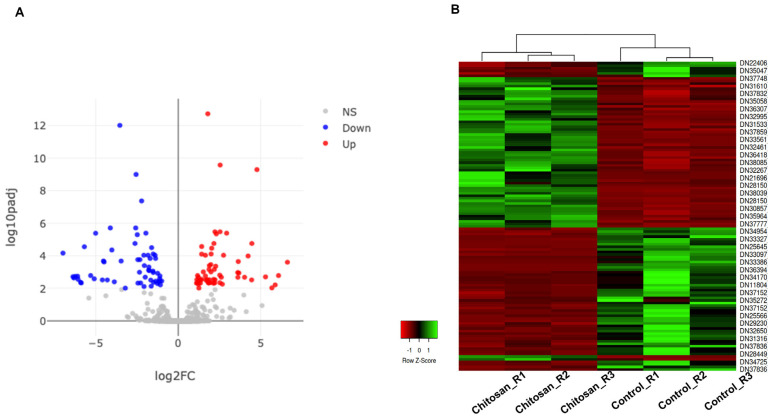
Visualization of significant differentially expressed genes (DEGs) in response to chitosan treatment. (**A**) The volcano plot displays 121 significant DEGs, which were divided into 60 down-regulated (blue dots) and 61 up-regulated (red dots). Gray dots indicate the remaining genes in which expression was not significantly changed. The representations are as follows: x-axis, logFC; y-axis, -log10 of the p-value. (**B**) The heat map shows the hierarchical clustering of 121 DEGs according to samples and genes. Up-regulated and down-regulated genes were shown in red and green bars, respectively.

To narrow down significant DEGs, we applied fold change > 2 and adjusted *p-value* < 0.01 as cutoffs resulting in 121 DEGs that were further divided into 61 upregulated and 60 downregulated genes (Table S1 and Figure 3A). For instance, representative highly upregulated genes were those encoding DMR6-LIKE OXYGENASE 2-like protein, MADS-box protein, DMR6-LIKE OXYGENASE 2-like protein, L-type lectin-domain containing receptor kinase IX.1-like protein, and terpene synthase 4 (Table S1). 

The highly downregulated genes were those encoding lectin 2a, low molecular mass early light-inducible protein HV90, COBRA-like protein, MFT-like protein, reticulon-like protein B10, C2 domain-containing protein, and PAR1-like protein (Table S1). Hierarchical clustering using the 121 DEGs was conducted in six different samples and shows two distinct groups of samples and two groups of genes (Figure 3B). 

### 2.4. Functional Annotation and Classification of Assembled Transcripts

**Figure 4:**
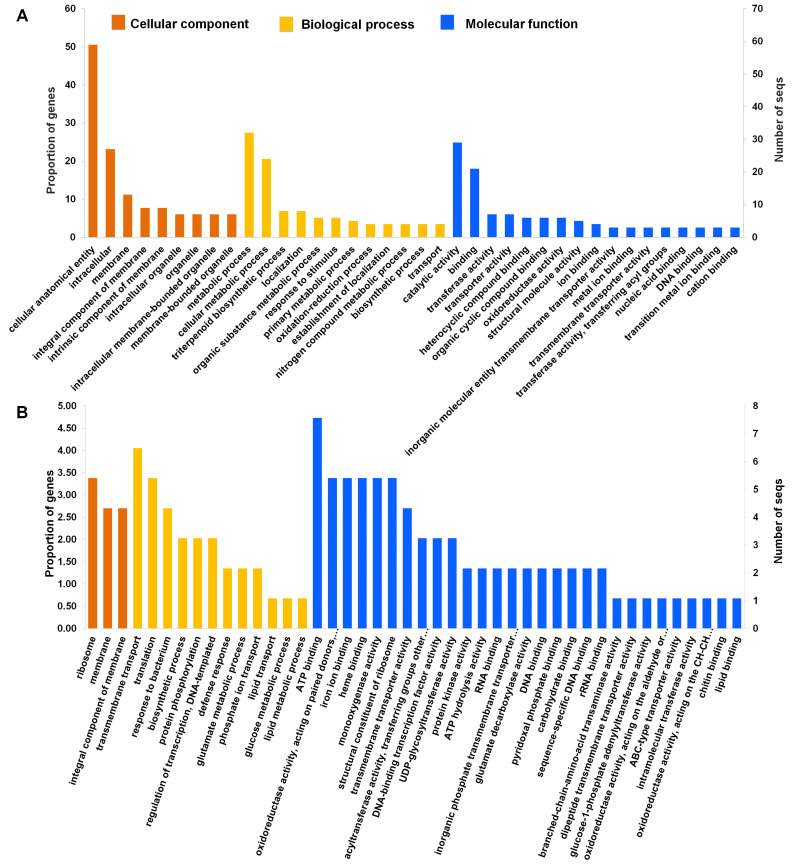
The number of enriched gene ontology (GO) terms in the identified DEGS. The number of identified GO terms according to cellular component (orange color), biological process (yellow color), and molecular function (blue color) in 3552 (**A**) and 121 (**B**) DEGs was visualized.

To further determine the enriched functions of the identified 3552 DEGs, we performed a gene ontology (GO) enrichment analysis using Fisher’s exact test with an FDR-adjusted *p-value* < 0.01 as the cutoff. All DEGs were functionally categorized based on GO terms according to three functional categories: biological process, cellular component, and molecular function. Significantly enriched GO terms were visualized (Table S2 and Figure 4A). We identified 18 enriched GO terms according to cellular components. Many DEGs were associated with a cellular anatomical entity (59 DEGs), intracellular (27 DEGs), membrane (13 DEGs), an integral component of membrane (9 DEGs), an intrinsic component of membrane (9 DEGs), which were found to be specific to secondary metabolite production. According to molecular function, 29 GO terms were enriched. Of these, GO terms associated with catalytic activity (29 DEGs), binding (21 DEGs), transporter activity (7 DEGs), and transferase activity (7 DEGs) were significantly enriched (Table S3 and Figure 4A). According to the biological processes, 139 GO terms were enriched and associated with secondary metabolites and plant defense mechanisms. For instance, the significantly enriched GO terms were associated with metabolic processes (32 DEGs), followed by cellular processes (27 DEGs), triterpenoid biosynthetic processes (eight DEGs), organic substance metabolic processes (six DEGs), cellular metabolic processes (five DEGs), primary metabolic processes (five DEGs), and oxidation-reduction processes (four DEGs). In addition, GO terms associated with responses to lipid and carbohydrate metabolic processes were significantly enriched (Figure 4A).

To obtain a more detailed insight into biological function, GO enrichment analysis was performed on a dataset of significant 121 DEGs. Using a threshold of false discovery rate (FDR) < 0.05, we found that many DEGs were targeted to three cellular components: ribosomes, membranes, and integral components of the membrane. According to the biological process, the top five enriched GO terms were associated with transmembrane transport, translation, response to bacteria, biosynthetic processes, and protein phosphorylation. The enriched GO terms according to molecular function were ATP binding, oxidoreductase activity, iron ion binding, heme binding, and monooxygenase activity.

Next, we performed an enrichment analysis against the Kyoto Encyclopedia of Genes and Genomes (KEGG) pathway to identify the key metabolic pathways enriched in the DEGs. All identified DEGs were assigned to at least one KEGG pathway and categorized into 236 metabolic pathways (Table S3). Of these, 38 pathways such as ABC transport, starch and sucrose metabolism, metabolic pathway, amino sugar and nucleotide sugar metabolism, and MAPK signaling pathway were significantly overrepresented under chitosan treatment (Table S3).

### 2.5. Metabolic Pathway Analysis and Identifications of Genes Involved in 20E Biosynthetic Pathways

Of the 3552 DEGs, 148 were involved in 21 biosynthetic pathways of secondary metabolites. We found that the most significant secondary metabolic pathways were monoterpenoid biosynthesis (48 DEGs); steroid and brassinosteroid biosynthesis (34 DEGs); triterpenoid, diterpenoid, and sesquiterpenoid biosynthesis (32 DEGs); and phenylpropanoid biosynthesis (24 DEGs). Of the DEGs involved in secondary metabolite biosynthesis pathways, we identified several transferases, including 2-C-methyl-D-erythritol-4-phosphate cytidylyltransferase (ispD), sterol 24-C-methyltransferase (ERG6), and 24-methylenesterol C-methyltransferase, as well as reductases, such as 7-dehydrocholesterol reductase (DHCR) and hydroxymethylglutaryl-CoA reductase (HMGR). We found that these transferases and reductases are involved in 20E biosynthesis in *Microsorum*.

**Figure 5:**
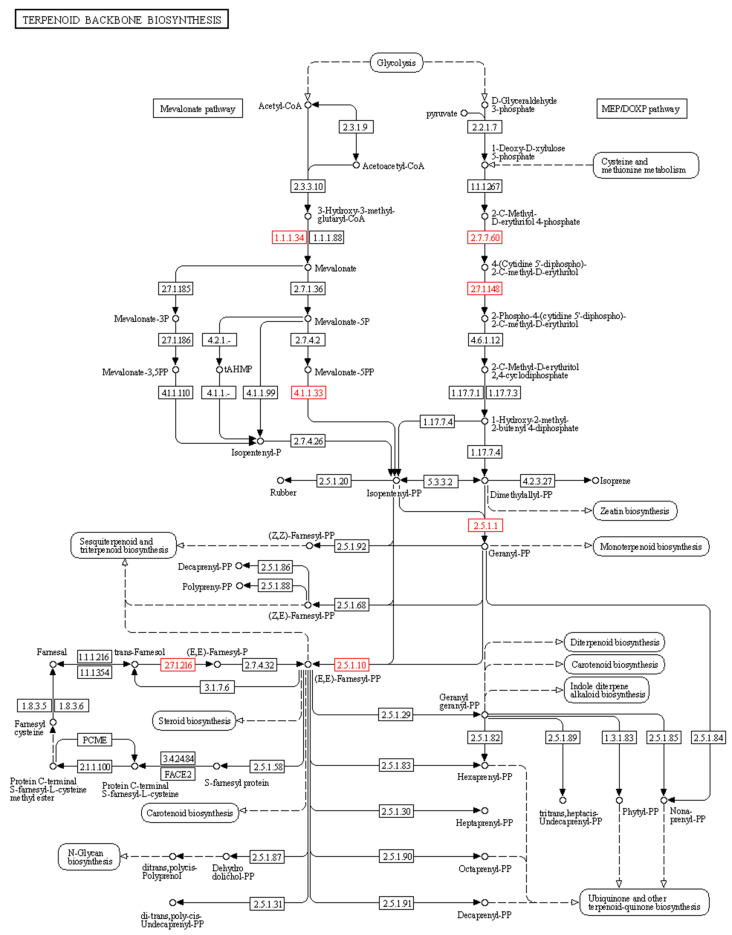
Key enzymes involved in terpenoid backbone biosynthesis. The identified DEGs encoding enzymes were highlighted by red colored boxes: (i) hydroxymethylglutaryl-CoA reductase (E1.1.1.34), (ii) diphosphomevalonate decarboxylase (E4.1.1.33), (iii) 2-C-methyl-D-erythritol 4-phosphate cytidylyltransferase (E2.7.7.60), (iv) 4-diphosphocytidyl-2-C-methyl-D-erythritol kinase (E2.7.1.148), (v) farnesyl diphosphate synthase (E2.5.1.1), (vi) farnesyl-diphosphate synthase (E2.5.1.10), and (vii) farnesol kinase (E2.7.1.216).

The compound 20E is a sesquiterpenoid phytosterol synthesized via terpenoid backbone biosynthesis. The KEGG pathway analysis in our study revealed 18 significant DEGs associated with the steroid biosynthesis pathway and terpenoid biosynthesis, leading to the downstream synthesis of 20E. These genes encode enzymes required for both the mevalonate (MVA) and methylerythritol 4-phosphate (MEP) pathways of terpenoid backbone biosynthesis (Figure 5). Several previous reports have shown crosstalk between both pathways in the synthesis of the secondary metabolite, 20E. For example, two DEGs encoding hydroxymethylglutaryl-CoA reductase (E1.1.1.34) and diphosphomevalonate decarboxylase (E4.1.1.33) in the MVA pathway have been identified. Moreover, DEGs encoding 2-C-methyl-D-erythritol 4-phosphate cytidylyltransferase (E2.7.7.60) and 4-diphosphocytidyl-2-C-methyl-D-erythritol kinase (E2.7.1.148), which are involved in the MEP pathways, were identified. Moreover, three additional DEGs encoding farnesyl diphosphate synthase (E2.5.1.1), farnesyl-diphosphate synthase (E2.5.1.10), and farnesol kinase (E2.7.1.216), which are required for monoterpenoid, sesquiterpenoid, and triterpenoid biosynthesis, were identified (Table S3 and Figure 5). 

**Figure 6:**
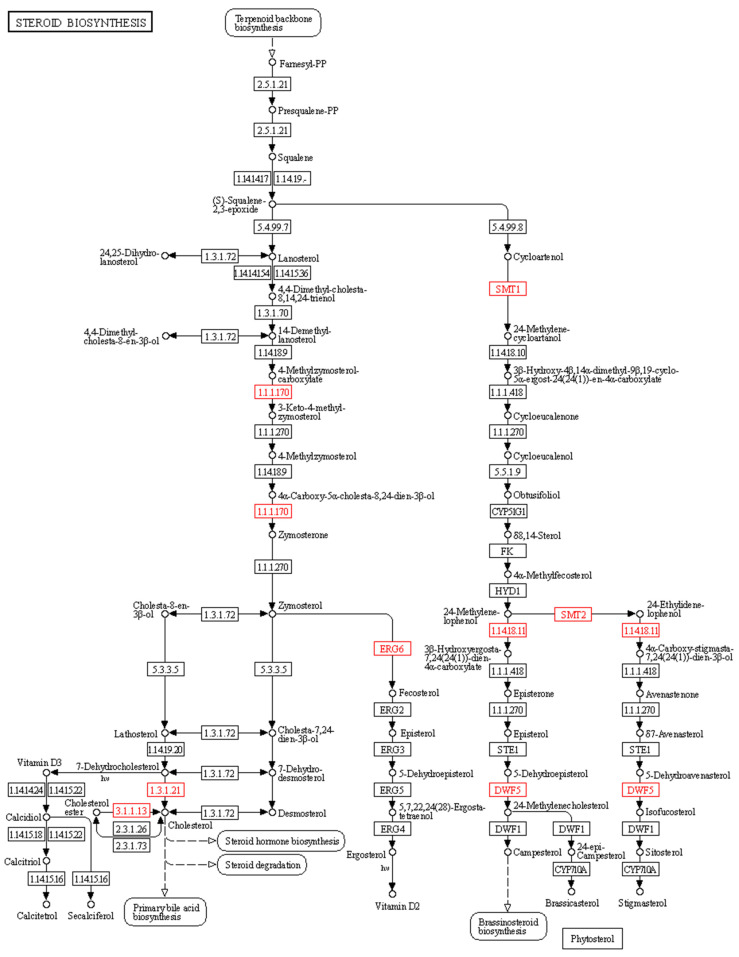
Key enzymes involved in steroid and phytosterol biosynthetic pathways. The identified DEGs encoding enzymes were highlighted by red colored boxes: (i) sterol-4alpha-carboxylate 3-dehydrogenase (E1.1.1.170), (ii) 7-dehydrocholesterol reductase (E1.3.1.21), (iii) cholesteryl ester hydrolase (E3.1.1.13) and sterol 24-C-methyltransferase (ERG6) (E2.1.1.41), (iv) diphosphomevalonate decarboxylase (E4.1.1.33), (v) 24-methylenesterol C-methyltransferase (SMT2) (E2.1.1.143), (vi) 4alpha-monomethylsterol monooxygenase (E1.14.18.11), and (vii) 7-dehydrocholesterol reductase (DWF5) (E1.3.1.21).

We also found that several DEG-encoding enzymes are involved in the steroid and phytosterol biosynthetic pathways for 20E synthesis (Figure 6). For example, we identified several DEGs encoding eight enzymes in the steroid biosynthesis pathway, mainly enriched in the cholesterol and phytosterol biosynthetic pathways. Moreover, we identified several DEGs encoding sterol-4alpha-carboxylate 3-dehydrogenase (E1.1.1.170), 7-dehydrocholesterol reductase (E1.3.1.21), cholesteryl ester hydrolase (E3.1.1.13), and sterol 24-C-methyltransferase (ERG6) (E2.1.1.41), which are required for the cholesterol pathway. Furthermore, we identified DEGs encoding diphosphomevalonate decarboxylase (E4.1.1.33), 24-methylenesterol C-methyltransferase (SMT2) (E2.1.1.143), 4alpha-monomethylsterol monooxygenase (E1.14.18.11), and 7-dehydrocholesterol reductase (DWF5) (E1.3.1.21) required for the phytosterol biosynthetic pathway (Table S3 and Figure 6). 

**Figure 7:**
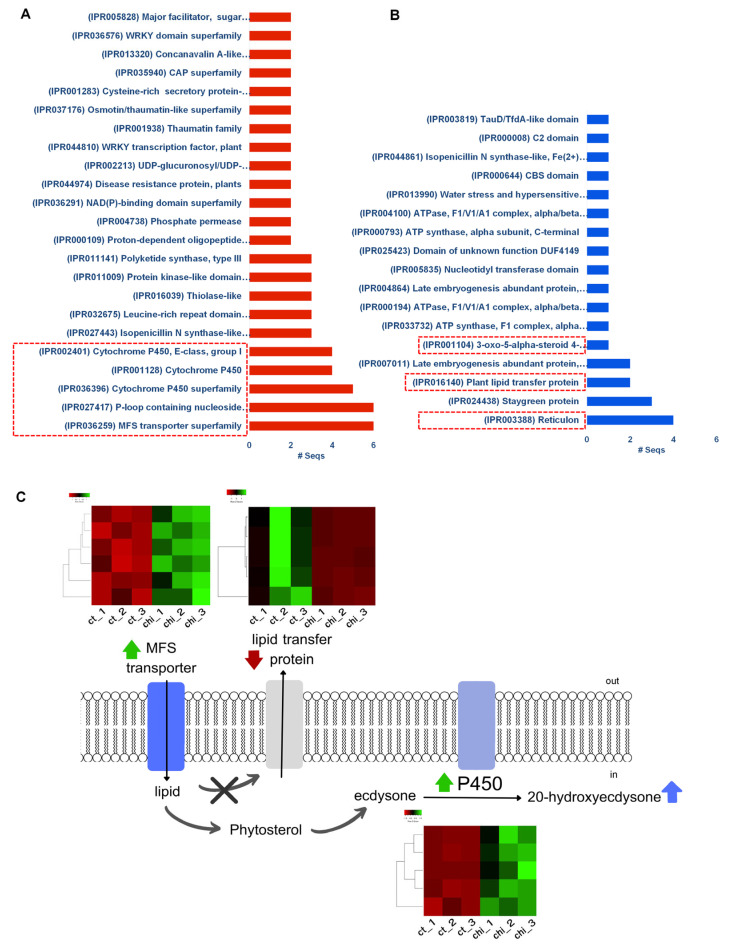
Enriched protein domains in the identified DEGs according to InterPro database. The enriched protein domains in the up-regulated genes (**A**) and down-regulated genes (**B**). The highlighted red boxes represent the functional domains of DEGs encoding transporter, signal transduction, and cytochrome P450. Proposed model for the effect of foliar spraying of chitosan on 20E elicitation in sterile fronds of *M. scolopendria* (**C**). Heatmaps showing the FPKM of genes involved in MFS transporter, lipid transfer protein, and cytochrome P450s within chitosan-treated Microsorum compared with the control. Upregulated and downregulated genes are indicated by green and red bars, respectively.

The functional domains of the proteins encoded by the upregulated genes were classified according to the InterPro database. As a result, a total of 41 protein domains were significantly enriched. The most enriched functional categories were transporter, signal transduction, and cytochrome P450 domains (Figure 7A). In contrast, only 17 protein domains were identified in downregulated genes. They were reticulon, stay-green protein, and plant lipid transfer protein domains (Figure 7B). Additionally, several transcription factors belonging to the AP2/ERF, MYB, and WRKY families were identified, which are involved in plant development and lipid metabolism (Table S3 and Figure 7). Many DEGs were also associated with transmembrane transport and ATP binding (Figure S1). Notably, numerous genes (51 DEGs) encoding cytochrome P450s were identified (Table S3). Based on functional annotation, various cytochrome P450s (cytochrome P450 71, 72, 83, and 709) related to specialized triterpene metabolism were identified (Table 2 and Table S3). All the DEGs encoding cytochrome P450s were up-regulated (Figure S1). The expression levels of CYP72A15 (2.164), CYP709B2 (2.227), CYP83B1 (4.452), and CYP71A1 (5.679) increased after chitosan treatment (Table 1).

### 2.6. Validation of RNA-seq Results Using Quantitative Real-Time RT-PCR

**Figure 8:**
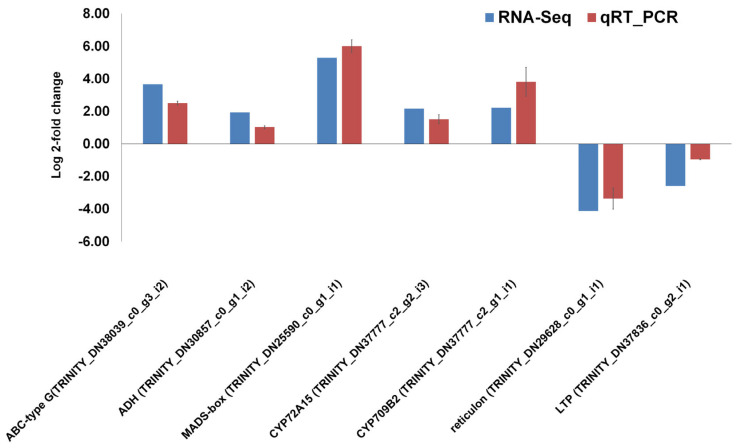
Validation of RNA-seq results using qRT-PCR. The fold changes of seven selected DEGs quantified using RNA-seq and qRT-PCR were shown. The blue bars represent RNA-seq results, and the red bars represent qRT-PCR results.

The seven DEGs including cytochrome P450 genes (CYP72A15 and CYP709B2), triterpenoid biosynthetic genes (ADH and MADS-box), ABC transporter-type G gene, and biosynthetic process-related genes (reticulon and LTP) were selected for the validation of RNA-seq results using quantitative real-time RT-PCR. We found that the expression tendency of all examined genes was consistent between the RNA-seq and qRT-PCR results. For example, genes encoding ABC-type G, ADH, MADS-box transcription factor, CYP72A15, and CYP709B2 were upregulated, whereas two genes encoding reticulon and LTP were downregulated (Figure 8).

## 3. Discussion

The detectable amounts of 20E in plants are very low, accounting for 1–2% of the dry weight in the few identified high-accumulators, whereas most 20E-accumulating species contain only 0.01–0.1% [27]. The 20E content must be considered not only when selecting a high-producing genetic cultivar, but also when considering treating the plants with appropriate elicitors. In this study, we examined the effects of foliar spraying of chitosan on the physio-biochemical and molecular responses of plants. Our results showed that all three concentrations (50, 100, and 200 mg/mL) of chitosan significantly (*p <* 0.05) affected the growth of *M*. *scolopendria.* Of the three different concentrations of chitosan, 50 mg/mL showed the highest increase in RGR in young and mature sterile fronds at 7 DAT. Moreover, chitosan treatment provided the highest growth-stimulating effect in young sterile fronds compared with that of the control. Natural polysaccharides, such as chitosan and its derivatives, improved the growth attributes and efficiency of uptake of individual elements, which in turn is associated with the intensification of plant growth [28,29]. Several previous studies have shown that chitosan treatment at lower concentrations results in increased vegetative growth in marigold, grape, okra, freesia, basil, and strawberry [30,31,32,33,34,35]. Moreover, the foliar application of chitosan inhibited the transpiration and gaseous exchange and increased the absorption of water and principal nutrients, thus providing water to biomass and yield. For example, the foliar application of chitosan solution to *Dracocephalum kotschyi* resulted in a significant increase in biomass [18,36]. Our study confirmed previous findings and validated that the application of chitosan promotes plant growth. Furthermore, our results demonstrated that elicitor concentration and timing of application might be very important in determining the optimal effects of chitosan.

Interestingly, foliar application of chitosan improved 20E accumulation in the sterile fronds of *M. scolopendria* compared with that in the untreated control. Chitosan foliar spray in common rue plants led to the accumulation of various types of secondary metabolites such as coumarins, acridone, quinolone alkaloids, and flavonoids [37]. Chitosan application is an effective elicitor for improving rosmarinic acid, quercetin, and apigenin levels up to 16-fold higher than that of the control [18]. Many investigators have reported the properties of chitosan in plant growth induction and secondary metabolite elicitation. However, only a few studies provide limited data on the effects of spray application of chitosan in the fern *M. scolopendria*, which remarkably increased the active 20E content in sterile fronds. This study shows that low doses of chitosan (50 mg/mL) sprayed on the fronds of *M. scolopendria* increased growth with a concurrent significant enhancement of 20E content in sterile fronds.

Transcriptome analysis of mature sterile fronds of *M. scolopendria* has been carried out as the fronds are the primary site of synthesis and accumulation of bioactive 20E. Chitosan is the main signal of secondary metabolite production across the plant kingdom, from angiosperms to tracheophytes. Chitosan treatment triggers the biosynthesis of the majority of secondary metabolites (i.e., terpenoids and phenylpropanoids) through extensive transcriptional reprogramming. The elicitation of secondary metabolite 20E enriched in *M. scolopendria* might be due to the role of chitosan in gene regulation and signaling, which leads to the activation of enzymes in metabolic pathways linked with 20E biosynthesis. In this study, using RNA-seq, we investigated transcriptional changes in *M. scolopendria* in response to 50 mg/mL chitosan. As a result, we narrowed the number of DEGs from 3552 to 121 ones. Similarly, several significant DEGs have been identified in response to chitosan in potato leaves, suggesting that the activation of a set of conserved genes positively influences plant growth, development, and metabolism [38]. Chitosan treatment induced the expression of 83 DEGs, most of which are involved in biological processes that promote photosynthesis and respiration. When we compared the RNA-seq data of the chitosan-treated ferns and potatoes, transcripts encoding mitochondrial proteins were shown to distinguish between these two treated plants, such as the genes encoding cytochrome c oxidase and ATP synthase subunits, which were upregulated in potatoes [38], whereas cytochrome c oxidase was not influenced by chitosan, and ATP synthase subunits were downregulated in *M. scolopendria*.

The identification of candidate genes and key enzymes is crucial for understanding the biosynthetic pathways of active 20E in *M. scolopendria*. 20E is a terpenoid synthesized from the terpenoid backbone biosynthesis pathway, which includes both MEP and MVA pathways [39]. The precursor of 20E biosynthesis operates more readily and intracellularly by following the plastidial MEP pathway and cytosolic MVA pathway, respectively [10]. Based on the enriched GO terms according to cellular components, both the plasticidal MEP and cytosolic MVA pathways were functional for the biosynthesis of 20E in *Microsorum*. Moreover, analyses of genes against the KEGG pathway revealed that many of the identified DEGs were involved in 236 metabolic pathways. They encode various enzymes and proteins involved in the biosynthesis of different isoprenoids such as monoterpenes, diterpenes, triterpenes, sesquiterpenoids, and steroids. 

A previous report also predicted that the major role of the terpenoid backbone biosynthesis pathway is specialized secondary metabolite biosynthesis [40]. The MVA pathway generally supplies the precursors for the production of sesquiterpenes, triterpenes, and brassinosteroids. The MEP pathway generally supplies the precursors for the biosynthesis of diterpenoids [41]. Identification of genes encoding 3-hydroxy-3-methylglutaryl coenzyme A reductase (HMGR) in this study correlated with an earlier study demonstrating that this enzyme is the rate-limiting enzyme of the MVA and cholesterol pathways. Overall, four HMGR genes were identified in the mature sterile fronds of *M. scolopendria* after foliar chitosan treatment. We noticed the involvement of HMGR in MVA. Moreover, cholesterol induced by chitosan treatment was required for the upstream processes of 20E biosynthesis. Additionally, we identified transferases, such as 2-C-methyl-D-erythritol-4-phosphate cytidylyltransferase (ispD), sterol 24-C-methyltransferase (ERG6), 24-methylenesterol C-methyltransferase, and reductases, such as 7-dehydrocholesterol reductase (DHCR) and hydroxymethylglutaryl-CoA reductase (HMGR). Genes associated with the triterpenoid biosynthesis pathway have frequently been identified in several medicinal plants [42]. Moreover, genes encoding key enzymes involved upstream of the 20E biosynthetic pathway, such as steroid 17α-monooxygenase, farnesyl diphosphate synthase, farnesol kinase, prenylcysteine α-carboxymethylesterase, diphosphomevalonate decarboxylase, 4-diphosphocytidyl-2-C-methyl-D-erythritol kinase, and 2-C-methyl-D-erythritol 4-phosphate cytidylyltransferase were identified in the present study. 

We identified 64 genes encoding ABC transporters. Genes in the ABC transporter family, based on the hydrolysis of ATP, are known to play a major role in the transport of secondary metabolites [43,44]. Since many plant secondary metabolites are used medicinally, ABC transporters are also associated with drug resistance [45]. For example, the indirect role of ABC transporters in regulating secondary metabolism has been studied by overexpressing enzymes involved in the primary metabolism [46]. Meanwhile, an inspection of the leaf epidermis enriched transcription database identified an ABC transporter that mediates the transport of anticancer drug components in the Catharanthus [45]. Therefore, ABC transporters may be associated with the transport of special compounds, including alkaloids, terpenoids, and polyphenols [47], and the secondary metabolite 20E. 

We identified several transcription factors involved in plant development and lipid metabolism including AP2/ERF, MYB, and WRKY. The ethylene responsive factor (AP2/ERF) superfamily and R2R3-MYB family belong to one of the largest diverse families of transcription factors and are involved in controlling plant lipid metabolism processes, biosynthesis of primary and secondary metabolites, and adaptation to stress [48]. Previous studies have shown that WRKY and ERF transcription factors regulate sterol synthesis in plants. WRKY1 belongs to the WRKY TFs and participates in regulating the levels of secondary metabolites such as sterols and glycoalkaloids in plants. For example, *WsWRKY1* silencing resulted in the hindrance of plant growth and decreased plant sterol content, whereas the overexpression of *WsWRKY1* promoted the synthesis of plant sterols in tobacco and tomato [49]. Furthermore, with the overexpression of *PqWRKY1* in Arabidopsis, the expression of plant sterol synthesis-related genes (such as *HMGR, FPS2, SQS1*, and *SQE2*) in transgenic lines increased one- to five-fold compared with that in the control [50]. 

Several upregulated genes encode amino acid transporters, which are essential for many substrates in plants and are involved in shuttling many secondary metabolite intermediates between subcellular compartments. The cytochrome P450 superfamily mediates the oxidation of various substrates involved in both primary and secondary metabolism, which enhances 20E accumulation in chitosan-treated *M. scolopendria* [51,52]. Recently, 52 genes of the cytochrome P450s family have been identified, which play crucial roles in terpenoid biosynthesis in various medicinal plants. The majority of these belong to the CYP71 family [53]. The most downregulated genes encoded members of the reticulon-like protein family and glutamate dehydrogenase (Table S4). In plants, reticulons are integral ER membrane proteins that are often associated with lipid synthesis, which might have a direct negative influence on 20E biosynthesis in *M. scolopendria* [54]. 

Based on gene annotation analysis of all DEGs, low concentrations (50 mg/mL) of chitosan application act as plant signal molecules to improve plant growth by inducing secondary metabolite 20E accumulation in the medicinal fern *M. scolopendria*. This process might occur via several mechanisms, such as (1) triggering various important transporter-mediated membrane transport, such as ATP-binding cassette (ABC) transporters and major facilitator superfamily (MFS) transporters, and repression of lipid transfer proteins that play a role in lipid transfer across the cytoplasm in plants (we suppose that these transporters play important roles linked to chitosan application in 20E biosynthesis by transporting substrate primary metabolites, especially lipid and terpenoid) and (2) stimulating plant steroid biosynthesis via highly expressed cytochrome P450s genes for a role in 20E biosynthesis. A previous study screened the five most highly expressed cytochrome P450 genes for phytoecdysteroid biosynthesis in the hairy roots of Ajuga [55]. The proposed model for the influence of chitosan on 20E elicitation is depicted in Figure 7C.

In conclusion, chitosan treatment increased the dry weight of the plant and 20E accumulation in *M. scolopendria*. RNA sequencing and DEG analyses revealed key enzymes that might be related to the production of the secondary metabolite 20E in *M. scolopendria*. We believe that the identified key genes associated with chitosan signaling and 20E production in *M. scolopendria* could be useful for further genetic analysis of 20E biosynthesis and pathway engineering.

## 4. Materials and Methods

### 4.1. Plant Materials and Foliar Chitosan Application

The fern *M. scolopendria* (Burm f.) Copel was grown in compost soil without fertilizer and under natural conditions in the experimental plot in a greenhouse at Kasetsart University, KamphaengSaen campus, Nakhon Pathom, Thailand. Each pot was arranged at a density of two pots per square meter. Chitosan from shrimp shells with an average molecular weight of 100 kDa (85% deacetylated chitin) (Sigma-Aldrich, Saint Louis, MO, USA) was dissolved in 5% acetic acid and diluted in distilled water to three different concentrations (50, 100, and 200 mg/mL). The pH of the chitosan solutions was adjusted to 6.5 with 2 M NaOH. Two months after transplantation, the plants were separated into four groups and arranged in a completely randomized block design with three replicates. One untreated group was designated as the control plants, whereas the remaining three groups were sprayed with chitosan solutions of various concentrations using an ultra-low volume sprayer. The spray application of chitosan on fully expanded fronds was performed in the early morning of every alternate day and continued for two weeks. The number of spray shots per plant was maintained at approximately 100 mL per pot. Young and mature sterile fronds were collected on day 0 after treatment and at 7 and 14 DAT. The fronds were rinsed with distilled water and dried at 60 °C until a constant weight was achieved. For transcriptome analyses, mature sterile fronds were sampled, immediately frozen, and stored at –80 °C. 

### 4.2. Plant Growth Measurement

Plant growth was measured based on the fresh and dry weights of the fronds. At three different time points (0, 7, and 14 DAT), three different concentrations of chitosan treatment (50, 100, and 200 mg/mL) were applied. Subsequently, young and mature sterile fronds were harvested and weighed before and after drying at 50 °C in a hot-air oven until a constant weight was achieved. Growth rates at consecutive treatment intervals were measured or estimated to determine the RGR. RGR (g g^−1^ d^−1^) was calculated as (*InW_2_ − InW_1_*)/(*t_2_ − t_1_*) for each individual *M. scolopendria*, where *W_1_* and *W_2_* are the mean dry mass of individual plants at time *t_1_* (the initial day of treatment) and time *t_2_* (the interval between treatments, days), respectively. 

### 4.3. Determination of 20-Hydroxyecdysone Content

The *M. scolopendria* plants were separated into two groups and arranged in a randomized block design with three replicates. One group was the control with no foliar spraying, whereas the other group was sprayed with 50 mg/mL chitosan using the method described earlier. Young and mature sterile fronds were harvested at 0, 7, and 14 d after chitosan treatment and dried in a forced-air oven at 50 °C until a constant weight was achieved. Dry fronds were powdered using a grinder and stored at –20 °C until extraction. The 20-hydroxyecdysone content was determined using a method described previously [1]. Dried tissue powder (200 g) was extracted with 2 L ethanol at 28–30 °C for 48 h. The supernatant was evaporated until the sample was dry, which was re-dissolved in methanol (5 mL). After filtration using a nylon membrane filter (0.45 μm) (Millipore, Darmstadt, Germany), aliquots of the reaction products (20 μL) were injected into a C18 reverse-phase column (4.6 mm × 250 mm) for HPLC analysis (Water Corporation, Miford, MA, USA). The mobile phase was a mixture of acetonitrile:propan-2-ol (5:2, *v*/*v*) or trifluoroacetic acid in water (20:80, *v*/*v*), at a flow rate of 1 ml min^−1^; UV detection was performed at a wavelength of 245 nm. Data, including the means of three replicates and ± standard error values, are presented as error bars.

### 4.4. Total RNA Extraction, cDNA Library Construction, and RNA Sequencing

RNA-sequencing was conducted to establish the transcriptional profiles of mature sterile fronds after treatment with 50 mg/mL chitosan at 14 DAT. Three replicates from each *M. scolopendria* frond treatment group were ground into powder in a mortar with liquid nitrogen, and total RNA was extracted from the samples using NucleoSpin^®^ RNA (MACHEREY-NAGEL, Düren, Germany) according to the manufacturer’s protocol. The quality and quantity of RNA were assessed using gel electrophoresis, an ND-1000 Nanodrop spectrophotometer (Thermo Scientific, Waltham, MA, USA), and an Agilent 2100 Bioanalyzer (Agilent, Santa Clara, CA, USA). DNA was removed from the samples using RNase-free DNase (Promega, Madison, WI, USA) according to the manufacturer’s instructions [56]. The RNA integrity number (RIN) values of these six samples ranged from eight to nine, which showed good quality for further downstream processes. After that, six cDNA libraries were generated for RNA-seq and paired-end sequenced using the NovaSeq 6000 platform. After removing the adapter sequences, duplicate sequences, ambiguous reads, and low-quality reads, clean reads between 22.18 and 30.74 million paired ends were generated (Table 2). cDNA synthesis was performed using equal quantities of high-quality RNA from each sample following TruSeq RNA Library Prep Kit v2 (Illumina, San Diego, CA, USA). Sequencing libraries were generated according to the manufacturer’s instructions (Illumina, San Diego, CA, USA). For paired-end sequencing, both ends of six cDNA libraries from three replications were sequenced using an Illumina NovaSeq 6000 system (Illumina, San Diego, CA, USA). The quantity and quality of the cDNA library were regulated by Macrogen (Seoul, Republic of Korea).

### 4.5. Assessment of Differential Gene Expression

Raw data obtained from the six samples were deposited in the Sequence Read Archive (SRA) database of NCBI with accession numbers: SRR8480631, SRR8480739, SRR8480744, SRR8481433, SRR8481526, and SRR8481655. High-quality reads were filtered by eliminating low-quality reads (Q < 20) from raw reads using BBDuk (https://jgi.doe.gov/data-and-tools/software-tools/bbtools/bb-tools-user-guide/bbduk-guide/ accessed on 1 December 2022). Filtered reads were used for mapping. The transcriptome of *M. scolopendria* from a previous study was used as a reference transcriptome [26]. Raw data were mapped to the reference transcriptome using the BBMap assembler with default parameters (https://jgi.doe.gov/data-and-tools/software-tools/bbtools/bb-tools-user-guide/bbmap-guide/ accessed on 1 December 2022). We obtained fragments per kilobase of transcripts per million mapped reads (FPKM) and mapped the read numbers for each transcript. DEGs were identified by comparing chitosan-treated samples (three samples) to non-treated samples (three samples). The mapped reads were used for DEG analysis using DESeq2 implemented in DEBrowser v1.24.1 [57]. Based on a fold change > two times and adjusted *p*-value < 0.05, we identified a total of 3552 DEGs in response to chitosan treatment. To identify significant DEGs, we used fold change > two times and adjusted *p*-value < 0.01 as cutoffs. The expression levels of the individual transcripts were quantified in terms of FPKM values.

### 4.6. Sequencing Annotation and Classification

We used annotated information for *M. scolopendria* transcriptome from a previous study [26]. For example, we evaluated several databases used previously to annotate *M. scolopendria* transcripts, including the National Center of Biotechnology Information (NCBI) non-redundant protein (NR, https://www.ncbi.nlm.nih.gov/protein/ accessed on 1 December 2022), Kyoto Encyclopedia of Genes and Genomes (KEGG) (http://www.genome.jp/kegg/ko.html accessed on 1 December 2022), Gene Ontology (GO, http://www.geneontology.org/ accessed on 1 December 2022), UniProt (http://www.uniprot.org/ accessed on 1 December 2022), and EggNOG (http://eggnogdb.embl.de/ accessed on 1 December 2022). GO terms were subsequently classified using Blast2GO [58] via OmicsBox with an e-value default cutoff. The enriched GO terms were identified according to three functional categories: biological processes (BP), cellular components (CC), and molecular functions (MF). For KEGG pathway analysis of the DEGs, bidirectional best hit (BBH) was used to search against the KEGG database to obtain the KO (reference pathway) number of KEGG annotations [59]. 

### 4.7. Validation of the Expression of Selected Genes Using qRT-PCR

We validated the RNA-seq results of seven selected genes involved in phytosterol biosynthesis using quantitative reverse transcription-polymerase chain reaction (qRT-PCR) analysis. Primers were designed using the Primer 3 software (www.embnet.sk/cgi-bin/primer3_www.cgi accessed on 1 December 2022). The primer names and sequences used for the primer design are listed in Table S5. Total RNA was extracted from each sample using TRIzol reagent (Invitrogen, Carlsbad, CA, USA) according to the manufacturer’s protocol. Total RNA was treated with DNase I (Thermo Scientific, Vilnius, Lithuania), and cDNA synthesis was performed using oligo(dT) primers and the RevertAid First Strand cDNA Synthesis Kit (Thermo Scientific, Dreieich, Germany). qRT-PCR was performed with 1 µL of cDNA in a total volume of 10 µL using iTaq Universal SYBR Green Supermix (Bio-Rad, Hercules, CA, USA) according to the manufacturer’s instructions. In detail, 2 µL of cDNA (1:10 diluted) was placed in a 20 µL reaction mixture containing 10 µL of iTaq Universal SYBR Green Supermix (2x) and 0.2 µL of each primer (10 pmol). qRT-PCR was run on a CFX96 qPCR machine (Bio-Rad, Hercules, CA, USA), and the thermal cycling conditions were adjusted as follows: 95 °C for 3 min, followed by 40 cycles of 95 °C for 10 s and 53 °C for 30 s. Actin was used as the reference gene to normalize the gene expression level, and relative gene expression was calculated using the Ct (2^−ΔΔCt^) method [60]. The experiment was performed with three biological replicates. Three technical duplicates were performed for every biological replicate. The outcomes of each biological replicate were calculated using the averages of the three technical replicates.

## Figures and Tables

**Table 1:** Identification of four differentially expressed genes (DEGs) associated with cytochrome P450s.

Transcript ID	Control_FPKM			Chitosan_FPKM			Log 2FC	Nelson’s P450 Name	Identity(%)	Length	e-Value
	1	2	3	1	2	3					
TRINITY_DN21696_c0_g1	1.029	0.955	0.929	12.340	18.233	33.000	4.452	CYP83B1	79.81	315	1.52 × 10 ^−43^
TRINITY_DN37777_c2_g2	18.517	14.321	15.785	51.417	80.023	86.000	2.164	CYP72A15	55.42	166	2.30 × 10^−58^
TRINITY_DN37777_c2_g1	22.632	20.050	18.571	63.757	122.567	100.000	2.227	CYP709B2	65.09	429	0
TRINITY_DN37987_c0_g7	0	0	0.929	5.142	17.220	31.000	5.679	CYP71A1	54.36	182	8.08 ×^−76^

**Table 2:** Summary of RNA-sequencing (RNA-seq) raw data. A total of six libraries were generated from *Microsorum scolopendria* untreated (three samples) and treated with chitosan (three samples). For each condition, three different libraries were paired-end sequenced using NovaSeq 6000 system. Raw sequence data were deposited in NCBI’s SRA database with the respective accession numbers.

Sample	Read Number	Base Number (bp)	GC Content (%)	Q20 (%)	Q30 (%)
control_R1	25,224,100	2,547,634,100	48.432	98.175	94.445
control_R2	23,174,306	2,340,604,906	48.362	98.222	94.573
control_R3	24,083,810	2,432,464,810	48.182	98.236	94.830
chtiosan_R1	23,169,198	2,340,088,998	48.870	98.099	94.356
chitosan_R2	22,181,024	2,240,283,424	48.519	98.325	94.769
chitosan_R3	30,743,418	3,105,085,218	48.910	98.246	94.631

## Data Availability

The raw dataset in this study is available in the Sequence Read Archive (SRA) repository with accession numbers SRR8480631, SRR8480739, SRR8480744, SRR8481433, SRR8481526, and SRR8481655.

## Supplementary Materials

The following supporting information can be downloaded at: https://www.mdpi.com/article/10.3390/ijms24032397/s1. Table S1: The list of differentially expressed genes (DEGs) between chitosan-treated and untreated *M. scolopendria* at 14 days after treatment (DAT); Table S2: The gene ontology (GO) classification of up-regulated and down-regulated DEGs; Table S3. The Fragments Per Kilobase of transcripts per million mapped reads (FPKM) value and Kyoto Encyclopedia of Genes and Genomes (KEGG) pathway annotation of DEGs; Table S4: The list of DEGs encoded for catalytic activity, transmembrane transport, ATP binding and cytochrome P450s; Table S5. Primers for quantitative real-time PCR; Figure S1: Heat map diagram representing transcript expression of DEG function in the most enriched GO categories such as catalytic activity, transmembrane transport, ATP binding, and cytochrome P450s.

## References

[1] Snogan E., Vahirua-Lechat I., Ho R., Bertho G., Girault J.P., Ortiga S., Maria A., Lafont R. (2007). Ecdysteroids from the medicinal fern *Microsorum scolopendria* (Burm. f.). Phytochem. Anal..

[2] Franco R., Takata L., Chagas K., Justino A., Saraiva A., Goulart L., Rodrigues V., Otoni W., Espindola F., Silva C. (2021). A 20-hydroxyecdysone-enriched fraction from *Pfaffia glomerata* (Spreng.) pedersen roots alleviates stress, anxiety, and depression in mice. J. Ethnopharmacol..

[3] Ho R., Teai T., Loquet D., Bianchini J.-P., Girault J.-P., Lafont R., Raharivelomanana P. (2007). Phytoecdysteroids in the Genus Microsorum (Polypodiaceae) of French Polynesia. Nat. Prod. Commun..

[4] Ambrosio G., Joseph J.F., Wuest B., Mazzarino M., de la Torre X., Diel P., Botrè F., Parr M.K. (2020). Detection and quantitation of ecdysterone in human serum by liquid chromatography coupled to tandem mass spectrometry. Steroids.

[5] Hunyadi A., Herke I., Lengyel K., Báthori M., Kele Z., Simon A., Tóth G., Szendrei K. (2016). Ecdysteroid-containing food supplements from Cyanotis arachnoidea on the European market: Evidence for spinach product counterfeiting. Sci. Rep..

[6] Dinan L., Dioh W., Veillet S., Lafont R. (2021). 20-Hydroxyecdysone, from Plant Extracts to Clinical Use: Therapeutic Potential for the Treatment of Neuromuscular, Cardio-Metabolic and Respiratory Diseases. Biomedicines.

[7] Wang W., Wang T., Feng W.-Y., Wang Z.-Y., Cheng M.-S., Wang Y.-J. (2014). Ecdysterone protects gerbil brain from temporal global cerebral ischemia/reperfusion injury via preventing neuron apoptosis and deactivating astrocytes and microglia cells. Neurosci. Res..

[8] Xia X., Zhang Q., Liu R., Wang Z., Tang N., Liu F., Huang G., Jiang X., Gui G., Wang L. (2014). Effects of 20-hydroxyecdysone on improving memory deficits in streptozotocin-induced type 1 diabetes mellitus in rat. Eur. J. Pharmacol..

[9] Shuvalov O., Fedorova O., Tananykina E., Gnennaya Y., Daks A., Petukhov A., Barlev N. (2020). An Arthropod Hormone, Ecdysterone, Inhibits the Growth of Breast Cancer Cells via Different Mechanisms. Front. Pharmacol..

[10] Savchenko R.G., Veskina N.A., Odinokov V.N., Benkovskaya G.V., Parfenova L.V. (2022). Ecdysteroids: Isolation, chemical transformations, and biological activity. Phytochem. Rev..

[11] Romaniuk A., Lisiak N., Toton E., Matysiak A., Nawrot J., Nowak G., Kaczmarek M., Rybczyńska M., Rubis B. (2021). Proapoptotic and proautophagic activity of 20-hydroxyecdysone in breast cancer cells in vitro. Chem.-Biol. Interact..

[12] Dioh W., Chabane M., Tourette C., Azbekyan A., Morelot-Panzini C., Hajjar L., Lins M., Nair G., Whitehouse T., Mariani J. (2021). Testing the efficacy and safety of BIO101, for the prevention of respiratory deterioration, in patients with COVID-19 pneumonia (COVA study): A structured summary of a study protocol for a randomised controlled trial. Trials.

[13] Schaller H., Liu H.-W., Mander L. (2010). 1.21—Sterol and Steroid Biosynthesis and Metabolism in Plants and Microorganisms. Comprehensive Natural Products II.

[14] Fujimoto Y., Maeda I., Ohyama K., Hikiba J., Kataoka H. (2015). Biosynthesis of 20-hydroxyecdysone in plants: 3β-hydroxy-5β-cholestan-6-one as an intermediate immediately after cholesterol in Ajuga hairy roots. Phytochemistry.

[15] Rogowska A., Szakiel A. (2021). Enhancement of Phytosterol and Triterpenoid Production in Plant Hairy Root Cultures—Simultaneous Stimulation or Competition?. Plants.

[16] Fooladi Vanda G., Shabani L., Razavizadeh R. (2019). Chitosan enhances rosmarinic acid production in shoot cultures of Melissa officinalis L. through the induction of methyl jasmonate. Bot. Stud..

[17] Khan T., Khan T., Hano C., Abbasi B.H. (2019). Effects of chitosan and salicylic acid on the production of pharmacologically attractive secondary metabolites in callus cultures of Fagonia indica. Ind. Crops Prod..

[18] Kahromi S., Khara J. (2021). Chitosan stimulates secondary metabolite production and nutrient uptake in medicinal plant Dracocephalum kotschyi. J. Sci. Food Agric..

[19] Lopez-Moya F., Lopez-Llorca L.V. (2016). Omics for Investigating Chitosan as an Antifungal and Gene Modulator. J. Fungi.

[20] Palma-Guerrero J., Lopez-Jimenez J.A., Pérez-Berná A.J., Huang I.C., Jansson H.B., Salinas J., Villalaín J., Read N.D., Lopez-Llorca L.V. (2010). Membrane fluidity determines sensitivity of filamentous fungi to chitosan. Mol. Microbiol..

[21] Suarez-Fernandez M., Marhuenda-Egea F.C., Lopez-Moya F., Arnao M.B., Cabrera-Escribano F., Nueda M.J., Gunsé B., Lopez-Llorca L.V. (2020). Chitosan Induces Plant Hormones and Defenses in Tomato Root Exudates. Front. Plant Sci..

[22] Kim Y.S., Cho J.H., Park S., Han J.-Y., Back K., Choi Y.-E. (2011). Gene regulation patterns in triterpene biosynthetic pathway driven by overexpression of squalene synthase and methyl jasmonate elicitation in *Bupleurum falcatum*. Planta.

[23] El Hadrami A., Adam L.R., El Hadrami I., Daayf F. (2010). Chitosan in plant protection. Mar. Drugs.

[24] Abdel-Rahman F.A., Monir G.A., Hassan M.S.S., Ahmed Y., Refaat M.H., Ismail I.A., El-Garhy H.A.S. (2021). Exogenously Applied Chitosan and Chitosan Nanoparticles Improved Apple Fruit Resistance to Blue Mold, Upregulated Defense-Related Genes Expression, and Maintained Fruit Quality. Horticulturae.

[25] Rudolf J.R., Resurreccion A.V.A. (2005). Elicitation of Resveratrol in Peanut Kernels by Application of Abiotic Stresses. J. Agric. Food Chem..

[26] Sripinyowanich S., Kil E.-J., Petchsri S., Jo Y., Choi H., Cho W.K., Lee S. (2021). De Novo Transcriptome Assembly of Two Microsorum Fern Species Identifies Enzymes Required for Two Upstream Pathways of Phytoecdysteroids. Int. J. Mol. Sci..

[27] Dinan L., Savchenko T., Whiting P. (2001). On the distribution of phytoecdysteroids in plants. Cell. Mol. Life Sci. CMLS.

[28] Ha N.M.C., Nguyen T.H., Wang S.-L., Nguyen A.D. (2019). Preparation of NPK nanofertilizer based on chitosan nanoparticles and its effect on biophysical characteristics and growth of coffee in green house. Res. Chem. Intermed..

[29] Salachna P., Pietrak A. (2021). Evaluation of Carrageenan, Xanthan Gum and Depolymerized Chitosan Based Coatings for Pineapple Lily Plant Production. Horticulturae.

[30] Akhtar G., Faried H.N., Razzaq K., Ullah S., Wattoo F.M., Shehzad M.A., Sajjad Y., Ahsan M., Javed T., Dessoky E.S. (2022). Chitosan-Induced Physiological and Biochemical Regulations Confer Drought Tolerance in Pot Marigold (*Calendula officinalis* L.). Agronomy.

[31] Khalil H.A., Eldin R.M.B. (2021). Chitosan Improves Morphological and Physiological Attributes of Grapevines Under Deficit Irrigation Conditions. J. Hortic. Res..

[32] Mondal M.M., Malek M., Puteh A., Ismail M., Ashrafuzzaman M., Naher L. (2012). Effect of foliar application of chitosan on growth and yield in okra. Aust. J. Crop Sci..

[33] Pirbalouti A.G., Malekpoor F., Salimi A., Golparvar A. (2017). Exogenous application of chitosan on biochemical and physiological characteristics, phenolic content and antioxidant activity of two species of basil (*Ocimum ciliatum* and *Ocimum basilicum*) under reduced irrigation. Sci. Hortic..

[34] Rahman M., Mukta J.A., Sabir A.A., Gupta D.R., Mohi-Ud-Din M., Hasanuzzaman M., Miah M.G., Rahman M., Islam M.T. (2018). Chitosan biopolymer promotes yield and stimulates accumulation of antioxidants in strawberry fruit. PLoS ONE.

[35] Salachna P., Zawadzińska A. (2014). Effect of chitosan on plant growth, flowering and corms yield of pottesd freesia. J. Ecol. Eng..

[36] Alavi Samany S.M., Ghasemi Pirbalouti A., Malekpoor F. (2022). Phytochemical and morpho-physiological changes of hyssop in response to chitosan-spraying under different levels of irrigation. Ind. Crops Prod..

[37] Orlita A., Sidwa-Gorycka M., Paszkiewicz M., Malinski E., Kumirska J., Siedlecka E.M., Łojkoswska E., Stepnowski P. (2008). Application of chitin and chitosan as elicitors of coumarins and fluoroquinolone alkaloids in *Ruta graveolens* L. (common rue). Biotechnol. Appl. Biochem..

[38] Lemke P., Moerschbacher B.M., Singh R. (2020). Transcriptome Analysis of Solanum Tuberosum Genotype RH89-039-16 in Response to Chitosan. Front. Plant Sci..

[39] Wang Y.C., Yang Y.Y., Chi D.F. (2018). Transcriptome analysis of abscisic acid induced 20E regulation in suspension Ajuga lobata cells. 3 Biotech..

[40] Rama Reddy N.R., Mehta R.H., Soni P.H., Makasana J., Gajbhiye N.A., Ponnuchamy M., Kumar J. (2015). Next generation sequencing and transcriptome analysis predicts biosynthetic pathway of sennosides from Senna (*Cassia angustifolia* Vahl.), a non-model plant with potent laxative properties. PLoS ONE.

[41] Rodrıguez-Concepción M., Boronat A. (2002). Elucidation of the methylerythritol phosphate pathway for isoprenoid biosynthesis in bacteria and plastids. A metabolic milestone achieved through genomics. Plant Physiol..

[42] Garg A., Agrawal L., Misra R.C., Sharma S., Ghosh S. (2015). Andrographis paniculata transcriptome provides molecular insights into tissue-specific accumulation of medicinal diterpenes. BMC Genom..

[43] Yazaki K. (2005). Transporters of secondary metabolites. Curr. Opin. Plant Biol..

[44] Yazaki K. (2006). ABC transporters involved in the transport of plant secondary metabolites. FEBS Lett..

[45] Fletcher J.I., Haber M., Henderson M.J., Norris M.D. (2010). ABC transporters in cancer: More than just drug efflux pumps. Nat. Rev. Cancer.

[46] Lloyd J.C., Zakhleniuk O.V. (2004). Responses of primary and secondary metabolism to sugar accumulation revealed by microarray expression analysis of the Arabidopsis mutant, pho3. J. Exp. Bot..

[47] Lv H., Li J., Wu Y., Garyali S., Wang Y. (2016). Transporter and its engineering for secondary metabolites. Appl. Microbiol. Biotechnol..

[48] Xing G., Li J., Li W., Lam S.M., Yuan H., Shui G., Yang J. (2021). AP2/ERF and R2R3-MYB family transcription factors: Potential associations between temperature stress and lipid metabolism in *Auxenochlorella protothecoides*. Biotechnol. Biofuels.

[49] Singh A.K., Kumar S.R., Dwivedi V., Rai A., Pal S., Shasany A.K., Nagegowda D.A. (2017). A WRKY transcription factor from Withania somnifera regulates triterpenoid withanolide accumulation and biotic stress tolerance through modulation of phytosterol and defense pathways. New Phytol..

[50] Sun Y., Niu Y., Xu J., Li Y., Luo H., Zhu Y., Liu M., Wu Q., Song J., Sun C. (2013). Discovery of WRKY transcription factors through transcriptome analysis and characterization of a novel methyl jasmonate-inducible PqWRKY1 gene from Panax quinquefolius. Plant Cell Tissue Organ Cult. (PCTOC).

[51] Kumar M.S., Babu P.R., Rao K.V., Reddy V.D. (2014). Organization and Classification of Cytochrome P450 Genes in Castor (*Ricinus communis* L.). Proc. Natl. Acad. Sci. India Sect. B Biol. Sci..

[52] Rao M.J., Xu Y., Tang X., Huang Y., Liu J., Deng X., Xu Q. (2020). CsCYT75B1, a Citrus CYTOCHROME P450 Gene, Is Involved in Accumulation of Antioxidant Flavonoids and Induces Drought Tolerance in Transgenic Arabidopsis. Antioxidants.

[53] Zhao Y.-J., Cheng Q.-Q., Su P., Chen X., Wang X.-J., Gao W., Huang L.-Q. (2014). Research progress relating to the role of cytochrome P450 in the biosynthesis of terpenoids in medicinal plants. Appl. Microbiol. Biotechnol..

[54] Chen J., Doyle C., Qi X., Zheng H. (2012). The endoplasmic reticulum: A social network in plant cells. J. Integr. Plant Biol..

[55] Tsukagoshi Y., Ohyama K., Seki H., Akashi T., Muranaka T., Suzuki H., Fujimoto Y. (2016). Functional characterization of CYP71D443, a cytochrome P450 catalyzing C-22 hydroxylation in the 20-hydroxyecdysone biosynthesis of Ajuga hairy roots. Phytochemistry.

[56] Laila R., Robin A.H.K., Yang K., Park J.-I., Suh M.C., Kim J., Nou I.-S. (2017). Developmental and genotypic variation in leaf wax content and composition, and in expression of wax biosynthetic genes in Brassica oleracea var. capitata. Front. Plant Sci..

[57] Kucukural A., Yukselen O., Ozata D.M., Moore M.J., Garber M. (2019). DEBrowser: Interactive differential expression analysis and visualization tool for count data. BMC Genom..

[58] Götz S., García-Gómez J.M., Terol J., Williams T.D., Nagaraj S.H., Nueda M.J., Robles M., Talón M., Dopazo J., Conesa A. (2008). High-throughput functional annotation and data mining with the Blast2GO suite. Nucleic Acids Res..

[59] Kanehisa M., Araki M., Goto S., Hattori M., Hirakawa M., Itoh M., Katayama T., Kawashima S., Okuda S., Tokimatsu T. (2007). KEGG for linking genomes to life and the environment. Nucleic Acids Res..

[60] Livak K.J., Schmittgen T.D. (2001). Analysis of relative gene expression data using real-time quantitative PCR and the 2−ΔΔCT method. Methods.

